# Embodying Language through Gestures: Residuals of Motor Memories Modulate Motor Cortex Excitability during Abstract Words Comprehension

**DOI:** 10.3390/s22207734

**Published:** 2022-10-12

**Authors:** Doriana De Marco, Elisa De Stefani, Giovanni Vecchiato

**Affiliations:** 1 Istituto di Neuroscienze, Consiglio Nazionale delle Ricerche, 43125 Parma, Italy; 2 Dipartimento di Medicina e Chirurgia, Università degli Studi di Parma, 43125 Parma, Italy; 3 Child and Adolescent Neuropsychiatry-NPIA District of Scandiano, AUSL of Reggio Emilia, 42019 Reggio Emilia, Italy

**Keywords:** embodied cognition, abstract language, TMS

## Abstract

There is a debate about whether abstract semantics could be represented in a motor domain as concrete language. A contextual association with a motor schema (action or gesture) seems crucial to highlighting the motor system involvement. The present study with transcranial magnetic stimulation aimed to assess motor cortex excitability changes during abstract word comprehension after conditioning word reading to a gesture execution with congruent or incongruent meaning. Twelve healthy volunteers were engaged in a lexical-decision task responding to abstract words or meaningless verbal stimuli. Motor cortex (M1) excitability was measured at different after-stimulus intervals (100, 250, or 500 ms) before and after an associative-learning training where the execution of the gesture followed word processing. Results showed a significant post-training decrease in hand motor evoked potentials at an early processing stage (100 ms) in correspondence to words congruent with the gestures presented during the training. We hypothesized that traces of individual semantic memory, combined with training effects, induced M1 inhibition due to the redundancy of evoked motor representation. No modulation of cortical excitability was found for meaningless or incongruent words. We discuss data considering the possible implications in research to understand the neural basis of language development and language rehabilitation protocols.

## 1. Introduction

How concrete and abstract concepts are represented in our brain?

Imagining the word “*run*” makes it easy to think about a prototypical action referred to it. On the other hand, the task grows harder when imagining the word “game”, considering the varieties of concrete experiences, situations, and contexts related to the same concept. 

Traditionally, concrete and abstract semantics were distinguished by the presence/absence of a relation of a concept with elements or actions present in the physical world. Concrete concepts can thus be processed by both a verbal-based and imagery-based system, while abstract words would land on a verbal-based system only [1]. 

However, recent research underlined the complexity and variety of abstract concepts, sustaining that they are not “detached” from the sensory world. Otherwise, they express flexible relationships with a multitude of “real-time” experiences processing, which involves multiple neural systems, including the sensorimotor one (for recent reviews, see [2,3,4,5,6,7]. 

Indeed, alongside “strong” language theories that are in favor of an obligatory involvement (“embodiment” theory, [8,9,10,11]) or a corollary involvement (“disembodiment” theory, [12]) of sensorimotor systems during language processing, new “extended” embodied approaches claim about a multidimensional representation of language. In this sense, language could be represented at the neural level in a multimodal way [13,14,15,16], involving motor representation at different levels, depending on the semantic content of each word and its degree of “grounding” in the physical world. In this perspective, the role of the sensorimotor system is considered modulable but not absent.

Some studies did not find traces of motor system involvement in response to abstract word processing [17,18,19,20,21]. However, more recent evidence demonstrated motor activation when abstract words were presented within a motor context, i.e., preceded by an action verb or a manual gesture [22,23,24,25,26]. The results of these studies may be explained as the effect of manipulating the association between the abstract word and a physical referent. This associational constraint “narrowed” the broader semantic representation of the abstract concept, enhancing motor activation. Nevertheless, additional studies found that the semantic processing of particular categories of abstract words (i.e., carrying emotional o mental content) recruits the motor system even if presented in isolation [27,28].

In attempting to explain these contrasting results, some authors proposed that differences in concrete and abstract language processing can be accounted for by different neural mechanisms that characterize the acquisition of different types of semantics [29,30,31]. Concrete or action language acquisition presumably goes through a directly experienced association between a signifier and a referent in the physical world. In contrast, abstract language learning is more mediated by social interaction during which verbal representations are shared (“WAT theory”, [32,33] or inferred through previously learned verbal associations [34].

One of the major lines of research on language acquisition is led by theories that postulate a motor origin of language development [35], as the gestural origin hypothesis [36]. Some authors proposed that spoken language functions rely on frontal brain regions, in particular in Broca’s area [37,38,39,40,41,42], where connections between hand and mouth evolved with the function of controlling feeding behavior [43,44]. Thus, the primary use of hand-arm gestures was enlarged to the mouth, inducing spoken language development through the gradual introduction of vocal elements associated with gestures [36]. Thenceforth, gestures (and, in general, hand movements) still accompany human communication exchanges [45], appearing as a precursor of the first attempts at speaking [46,47,48] and mediating language knowledge through sensory experience [49].

Studies demonstrating a tied integration in gesture and language processing (see [50,51,52,53] for a review) and the overlapping development of gesture and speech behavior in children [54,55] outlined a strong interconnection between actions, gestures, and language. Through gestures, the motor system participates in social communication with action pantomimes, iconic/deictic manual postures, and signs conventionalized across cultures. Symbolic gestures (i.e., hand or arm postures associated with a concept) give a clear example, being deliverable without the presence of speech though expressing a social-established meaning [56]. Symbolic gestures represent the best candidate to study the relationship between the motor system and language: they share the semantic properties with language, maintaining a manual representation, so being processed by the same systems involved in action understanding [42].

Given that gesture is considered an “evolutionary bridge” through language development, it is surprising that its role in abstract language processing and learning was poorly addressed.

In a transcranial magnetic stimulation (TMS) study, [26] assessed how gestures (primes) presented in a semantic priming paradigm influence the lexical-semantic processing of consequent abstract words (targets). When gestures and words were congruent in meaning, participants showed faster lexical decision times than conditions with pseudo-words targets or meaningless primes presentation. This result was associated with increased hand motor cortex (M1) excitability between 100 and 250 ms from word presentation, suggesting the motor system’s pivotal role in the earliest phase of language processing [18,57]. Moreover, the motor and behavioral facilitations reflected language integration with gesture meaning [51,52,58]. However, it is unclear if this effect represents temporary facilitation delimited to the condition of simultaneous processing or if it results from a beneficial, long-lasting impact of gestures in language learning.

The early studies investigating the latter hypothesis evidenced that enriching the verbal learning process with gesture performing favors the acquisition of a new abstract lexicon in humans ([59,60,61,62] and robots (i.e., numerical concepts, [63,64,65]). In this direction, defining manual actions as a crucial element in language learning could help to determine new paradigms to investigate the function of gesture in abstract lexicon development at the ontogenetical level.

The present study aims to assess how conditioning gesture execution within semantic processing modulates motor system involvement during abstract word comprehension. Second, we expected to evidence if motor response was affected or not by traces of previously learned gesture-word representations.

For this purpose, healthy participants were required to make a lexical decision about a series of meaningful words without any action or motor content presented with an equal number of meaningless words. Motor cortex excitability was assessed by delivering a single TMS pulse in correspondence to the hand motor cortex (M1) along with different delays from word presentation corresponding to pre-lexical (100–250 ms) and post-lexical (500 ms) phases of semantic processing. The same task was repeated after an associative-learning training where the presentation of each meaningful or meaningless word was coupled with the execution of a specific gesture, congruent or not with the previous words meaning.

Considering previous literature, we had two main hypotheses: (a) Gesture training could increase motor excitability in response to meaningful words, underlining specific motor representation related to each word’s meaning; and (b) this effect could be interfered with by previous common sensorimotor representation, i.e., the semantic relation between gestures and words, being specific for congruent words only.

## 2. Materials and Methods

### 2.1. Participants

Twelve participants (7 females, mean age of 21.5 ± 2.3 years) were enrolled in the experiment. All participants were right-handed (according to Edinburgh Handedness Inventory), [66], and Italian native speakers. They had normal or corrected-to-normal vision and no neurological or psychiatric disorder history. All participants provided written informed consent and declared no contraindication to applying single-pulse TMS [67,68]. The Ethics Committee of the Medical Faculty at the University of Parma approved the study.

### 2.2. Stimuli

Gestures stimuli were three pictures showing actress postures executing different symbolic gestures (thumb up, thumb down, and palm upward) and were presented in the training phase. Verbal stimuli were 12 pictures showing a still actress with opened mouth and a strip on the top on which a meaningful or meaningless Italian word was written (see Table 1). We selected half of the meaningful words, incongruent with gesture meaning, from an Italian database of abstract terms [69]. The other half of the words were chosen congruently with gesture meaning, matching them in length and occurrence with incongruent words (COLFIS database; [70]). Since these latter are not present in the Villani and colleagues database, we checked that the English translations are commonly classified as abstract concepts (Calgary semantic decision database, [71]). 

Meaningless words (Pseudowords) were matched in length with the corresponding meaningful stimuli (Congruent: *neba*, *lema*, *merfo*; Incongruent: *ritia*, *dife*, *or deia*). A further picture of a black cross on a grey background was presented randomly as control baseline stimuli.

### 2.3. Experimental Procedure

The experiment was carried out in a soundproofed room. Participants were seated in a comfortable armchair with their arms placed under a table plane. They were positioned on a head and chin rest to guarantee the stillness of their head throughout the TMS stimulation.

Stimuli were presented on a PC display 60 cm far from participants’ eyes. Stimuli presentation and TMS pulses were time-controlled through a script developed with Matlab software (Matlab version 7.7, R2008b; Psychophysics Toolbox extensions [72,73]).

**Figure 1:**
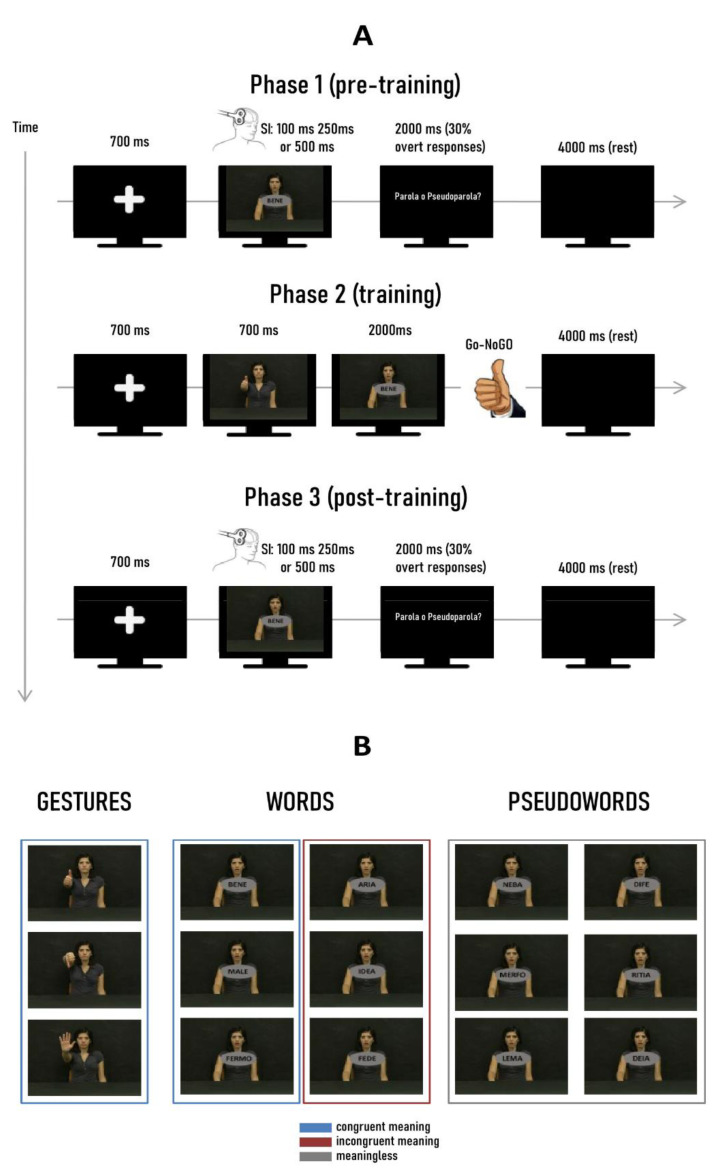
(**A**) Experimental paradigm: the experiment was divided into three phases: (1) pre-learning, (2) learning, and (3) post-learning. Pre-learning and post-learning procedures were identical. Participants observed a fixation crossed and had to make a lexical decision task in response to a verbal stimulus presentation (word or pseudoword). No gestural stimuli were presented in this phase. In the training phase (2), participants observed a gesture as a prime stimulus, followed by a meaningful verbal stimulus (congruent or incongruent with the gesture). Then, they had to perform the observed gesture (Go condition). Pseudowords were presented randomly as a No-Go condition that no required gesture execution. (**B**) Gestural and verbal stimuli presented in the experiment. Colored frames indicate the semantic relation of each class of verbal stimuli with gestures (congruent, incongruent, or meaningless).

The experiment was divided into three phases (Figure 1): phase 1 (PRE-TRAINING), phase 2 (TRAINING), and phase 3 (POST-TRAINING). Phase 3 was identical to phase 1 and was administered after the training to measure changes in motor cortical excitability.

In phases 1 and 3, each trial began displaying a fixation cross printed on a black screen (duration 700 ms), followed by the presentation of a verbal stimulus (word or pseudoword) which lasted 550 ms. A single TMS pulse on the left primary motor cortex (M1) was then delivered 100, 250, or 500 ms after verbal stimuli onset (stimulation delays). Each verbal stimulus was presented nine times for each stimulation delay (in total, 108 stimuli). Baseline trials were run randomly during phases 1 and 3, showing the cross instead of word/pseudoword and maintaining the same stimulation delays (in total, 27 trials). After the verbal stimulus was turned off, an interval of 4000 ms (black screen) interspersed the subsequent trial. Then, participants were required to perform a lexical-decision task classifying each verbal stimulus as a word or pseudoword. It is worth noting that they were unaware of the semantic relation between the observed word and the gestural stimuli presented in the training phase. The task was performed covertly except in 30% of the trials, where an overt response (button pressing with the left arm) was required in correspondence to a question mark appearing. Trials with incorrect answers or no responses were discarded for subsequent analysis.

In the intermediate training phase (phase 2), each trial started with the displaying of the fixation cross (700 ms duration), followed by the presentation of a gesture (duration 700 ms). Then, a verbal stimulus was presented and lasted 2000 ms. We did not administer TMS in this phase. Participants were required to make a lexical decision about the verbal stimulus and reproduce the presented gesture if the following word was meaningful (go trials), regardless of the congruent/incongruent semantic relationship.

Each congruent and incongruent word was matched with a specific gesture, forming the following couples (gesture—congruent word/gesture incongruent word: *thumb up*—*alright, thumb up*—*air; thumb down*—*badly, thumb down*—*faith; palm upward*—*tillness, palm upward*—*idea*). Each couple was repeated 40 times (in total, 240 trials). Additional 24 trials (10% of training trials) were administered, presenting pseudowords as linguistic stimuli (no-go trials). Thus, no pseudoword was associated with a gesture execution. 

### 2.4. Single-Pulse TMS Protocol and MEPs Recording

A single-pulse TMS was delivered to the left M1, and the corresponding MEPs were recorded from the right first dorsal interosseus (FDI). We acquired continuous electromyographic (EMG) recording from FDI with a CED Micro 1401 (Cambridge Electronic Design, Cambridge, U.K.) connected to CED 1902 amplifier and interfaced with CED Spike software. EMG signal was amplified (1000×), band-pass filtered (20–2000 Hz), and digitized at a sampling rate of 5 kHz through a PC software (Spike2, CED Ltd, Cambridge, UK). Pairs of surface electrodes (Ag–AgCl, disposable, 7 mm × 4 mm) were attached to the muscle belly (active electrode) and the corresponding metacarpophalangeal joints (reference electrode). The ground electrode was placed on the left wrist. The EMG signal was visualized and then processed offline. A figure-of-8 coil (Magstim Co., Ltd., Whitland, UK) connected to a Bistim system (Magstim Co., Ltd., Whitland, UK) was placed over the left M1. The coil intersection was placed tangentially to the scalp, with the handle pointing backwards and laterally, 45° angle away from the midline. The coil was moved to find the optimal position from which maximal amplitude MEPs were elicited in the contralateral FDI muscles using slightly supra-threshold stimulus intensity. We then marked the optimal position on the scalp to ensure correct coil placement throughout the experiment. The intensity of magnetic pulses was set at 120% of the resting motor threshold (RMT), defined as the minimal intensity of the stimulator output that produces MEPs with an amplitude of at least 50 μV in the muscles with a probability of 50% [68].

The experimenter visually inspected the absence of voluntary contraction throughout the experiment. When muscle tension was detected, participants were invited to relax. Trials with muscle contractions unrelated to TMS were discarded in the offline MEP analysis. MEPs of the FDI muscle were visually inspected and rejected if contaminated by contraction not due to stimulation (<3% of the total trials). The peak-to-peak amplitude (mV) was computed using MATLAB software (MATLAB R2016b). We discarded MEPs amplitudes less than 50 μV (<3% of the total trials) from the analysis. 

Raw MEPs amplitude of baseline trials has been checked for differences by means of a within-subject ANOVA with TRAINING (pre-training vs. post-training) and STIMULATION DELAY (T1, 100 ms vs. T2, 250 ms vs. T3, 500 ms). The normalized MEP amplitude of experimental trials was calculated for each subject by subtracting the individual baseline mean value from every raw MEP. Average normalized MEPs values calculated for each stimulation delay were submitted to a repeated-measures ANOVA with TRAINING (pre-training vs. post-training), SEMANTICS (pseudoword congruent vs. pseudoword incongruent vs. word congruent vs. word incongruent), and STIMULATION DELAY (T1, T2, T3) as within-subjects factors. All post-hoc comparisons were carried out using the Duncan test. Significance was established in all analyses at *p* = 0.05. The data’s normal distribution and sphericity were verified before performing statistical analysis (Mauchly’s test, *p* > 0.05; Kolmogorov–Smirnov Test, *p* > 0.05). η2 partial was calculated as a measure of effect size and 1-β as a measure of statistical power.

## 3. Results

Participants recognized above chance whether the presented stimuli were words or pseudowords. The mean percentage of correct responses was 98.1%.

Raw mean MEP amplitudes of FDI of baseline trials (cross pictures) administered during the experimental session were not significantly different from each other. No main effect nor significant interaction effects were found (*p* > 0.05).

The ANOVAs carried out on FDI normalized MEPs amplitude evidenced a significant interaction between SEMANTIC and STIMULATION DELAY factors (F(6,66) = 2.52, *p* = 0.029, η2 partial = 0.19, 1 − β = 0.80) and between TRAINING, SEMANTICS and STIMULATION DELAY (F(6,66) = 2.59, *p* = 0.026, η2 partial = 0.19, 1 − β = 0.82).

**Figure 2:**
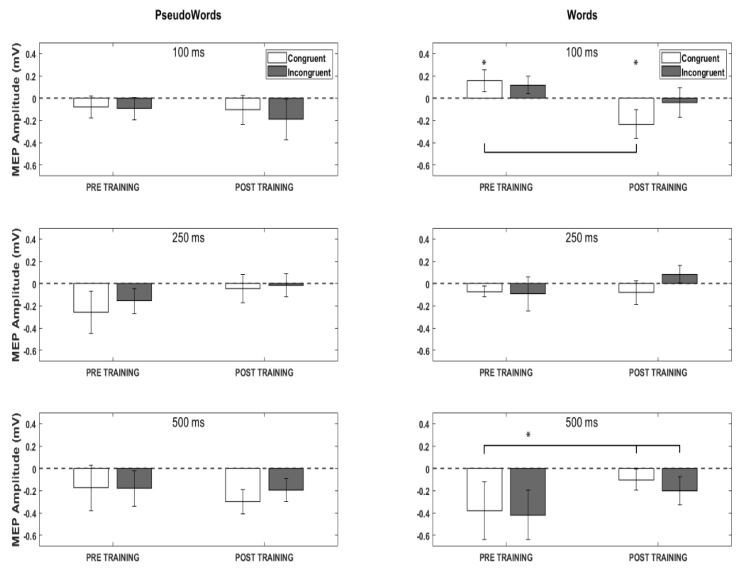
Averaged Motor evoked potentials (MEPs) of FDI muscle recorded after single-pulse TMS to hand primary motor cortex. Data are normalized to the baseline condition. MEPs values corresponding to T1 stimulation interval are presented in the upper row. Colored bars indicate the semantic relation between verbal stimuli and gestures. White bars represented data from congruent verbal stimuli, while black bars represented data from incongruent verbal stimuli. The middle and lower rows depict MEPs data corresponding to T2 and T3 stimulation intervals, respectively. Vertical and horizontal bars represent Standard Errors and significance in the statistical comparisons. Asterisks indicate significant *t*-test against zero (baseline values).

Post-hoc analysis evidenced a significant difference in hand M1 activation in response to congruent word stimuli before and after training at T1 stimulation delay (100 ms after word presentation) (*p* = 0.004). Indeed, while in the pre-training phase congruent words presentation significantly induced an augment of excitability, being the MEPs amplitude statistically different from baseline (t(11) = 2.56, *p* = 0.03), gesture training induced a motor inhibition (t(11) = 2.34, *p* = 0.03, Figure 2, upper-right). A further difference concerning Congruent Words was found in the correspondence of T3 stimulation delay (500 ms after word presentation) compared to T1 and T2 in the pre-training phase (*p* < 0.0001; *p* < 0.02). In this last stimulation interval, an opposite pattern emerged: starting from an average MEP value largely below the baseline, the hand M1 response to Congruent and Incongruent Words significantly increased after the training, revealing an augment of cortex excitability but showing significance only versus pre-training Congruent Words. However, despite this boost, cortical excitability remains below the baseline level. The same tendency also emerged for incongruent words without revealing significant differences. 

No significant results emerged concerning pseudowords.

A list of post-hoc results of the three factors’ interaction was presented in Table 2. Comparisons resulting with *p* > 0.05 and comparisons resulting in significant but mixing stimulation time and stimuli factors are not reported for simplicity and unuseful interpretability.

In sum, after the training, we observed a modulation of hand motor excitability specific for words that were semantically congruent with the associated performed gesture. 

Interestingly, MEPs re-measured in correspondence to the T1 stimulation interval showed a significant inhibition compared to baseline. At the post-semantic level of processing (T3 stimulation interval), we found an effect on training in increasing MEPs for the same class of words, even if the general excitability remains under baseline values. Conversely, we found no significant changes in MEPs for words that were equally associated with gestures during the training but conveyed an incongruent meaning or pseudowords (untrained stimuli).

## 4. Limitations

The small sample size represented a limitation of the study. To manage this issue, we computed a sensitivity analysis with GPower software (GPower 3.1, Universität Düsseldorf: Psychologie—HHU, Düsseldorf, Germany). The output of this analysis reported the theoretical acceptable minimum effect size given the alpha and 1-β obtained as the result of the ANOVA. This analysis permitted comparing the experimental and theoretical effect size to facilitate the interpretation of practical significance and the impact of the study results.

The sensitivity analysis computed concerning the interaction effect between TRAINING, SEMANTICS, and STIMULATION DELAY factors showed a minimum effect size of η2 partial of 0.09, relatively smaller than the obtained effect size (0.19). This result, in association with the large effect size and power values, offered a good statistical basis to sustain the significance and reproducibility of the results.

## 5. Discussion

Several studies evidenced the interaction between gestures and language during lexico-semantic processing and communication [50,51,52,58,74]. These findings support the hypothesis that gestures and speech are processed and integrated by the same neural systems involved in action perception and execution (mirror mechanism, see [75,76]).

However, even if a vast literature of behavioral, TMS, and neuroimaging studies has evidenced motor activation in response to action language understanding [18,19,20,21,77,78,79,80], evidence concerning abstract language is still contrasting.

In this study, we investigated if the effect of repeated gesture execution in association with the language comprehension process modulated the grade of motor representation, measuring motor system excitability in response to abstract words congruent or not in meaning with the performed gestures.

This paradigm was developed based on a previous study [26], where we found motor facilitation in response to abstract word presentation preceded by congruent gesture, demonstrating that both signals shared a motor representation that was retrieved to accomplish word understanding.

In the present study, we did not present any motor prime associated with the words. Symbolic gestures and words were matched through associative learning training to constrain the lexical-decision task to gesture processing and execution. In this way, we expected to sufficiently shape the motor response to word processing since the learned motor schema resulted automatically evoked within the lexical task.

The study results showed a significant change in hand motor cortex excitability in response to meaningful words related to gestures at T1 (100 ms after stimulus onset) between the pre-learning and post-learning phases. According to previous studies, this timing represents an automatic (bottom-up) phase of lexical-semantic elaboration [18,22,26,52,57]. As expected, residuals of previous learning (i.e., individual semantic memory) interfered with training effects. Indeed, the modulation of motor system response was limited to words with a congruent semantic relationship with the gestures presented during the training.

However, this modulation turned out to be a significant decrease as the effect of the training, in contrast with our original hypothesis that predicted higher motor facilitation consequent to the reinforcement of the semantic relation between each word and gesture. This result accords with a previous study by [50], where authors found inhibition of kinematic parameters when subjects performed gestures contemporary to the pronunciation of the corresponding-in-meaning word. The authors explained this result by claiming the overlap between the cerebral mechanisms associated with speech and manual activity (see [44]. In that case, parallel to a vocal spectra increase, the manual system was interfered with because of the involvement of the same brain process. Similarly, the motor inhibition found in our study could result from a conflict between overlapping neural activations. Previous neurophysiological studies showing an interference effect on semantic processing caused by the co-activity of the motor system corroborated this hypothesis [17,81,82]. The excitability decrease in the motor cortex would then represent the neural correlate of a motor response suppression. Since the combination of gesture and word during the training enhanced the motor representation related to the conveyed semantics, the following gesture execution added a probably redundant motor response.

The interesting datum is that the effect was evident only in the condition of words congruent with gestures, excluding the possibility that a general inhibitory control affected the motor response in the post-learning phase, where gesture execution was no more required.

An unexpected result concerns the increase of motor excitability observed in the pre-learning phase for congruent words compared to baseline values. The motor facilitation started 100 ms after stimulus onset and progressively decreased in the subsequent intervals. This result contrasts with a previous study [52], where we found no significant motor activation in response to gesture-congruent meaningful words compared to meaningless stimuli isolated from a gestural context.

We offer a possible explanation of this phenomenon considering the possible unbalance between the selected verbal stimuli in the present and past studies regarding valence. It is well-known that abstract semantics entail affective processing to a greater extent than concrete concepts [83,84], involving the precentral cortex and limbic regions [85]. A possibility is that in our study, the motor activation could be highlighted by the emotional value conveyed by the meaningful stimuli. Moreover, recent literature reports motor activation in response to specific abstract categories (e.g., numbers: [86]; Internal states: [87]; Aesthetics: [88]. Even if we cannot categorize our verbal stimuli in a single category, the presented words may engage, at least in part, the sensorimotor system.

Another result concerned the motor facilitation emerged at T3 (500 ms after stimulus onset): the MEPs in correspondence to congruent words significantly increased compared to the value recorded at the same time before the training, in the opposite tendency to the results of T1. In our opinion, this could be ascribed to a corollary activation of the motor system due to deeper semantic access that re-activates the motor content associated with the word, probably involving top-down processes (i.e., motor imagery). However, even if the modulation before and after training was significant, no significant augmenting of motor excitability beyond the physiological baseline appeared in the post-lexical phase of word comprehension.

In conclusion, the results of our study corroborated recent evidence which demonstrated the motor system’s role in abstract language processing. This is in line with revised embodied language theories, integrating the concept of a multimodal representation of the words [13,14,30,32,49,80]. Sensorimotor areas can be involved in the early phase of comprehension at different grades, depending on the contextual availability of motor schemas associated with them.

In this study, we used gestural stimuli to demonstrate how a learned association between a symbol (i.e., a word) with a motor posture shapes motor activation in a somatotopic way during a lexical-semantic task, interfacing with previous semantic knowledge of the subjects. These data suggest that the motor system may contribute through a similar mechanism to building semantic knowledge during language development in childhood, shaping and reinforcing specific neural pathways based on the relationship between a phoneme and a movement or physical entity [89]. This relation would develop depending on contextual variables and lexical flexibility [90], being sparser in abstract words due to the wide variety of meaning representations [5].

Further studies should clarify at which level concrete and abstract language could or not diverge in acquisition and processing at a behavioral and neural level.

A suggestion is to investigate the interaction between gestures and words following the action-language continuum [56]. While we easily understand pantomimes as mimed actions in association (or substitution) of action verbs, emblems comprehension results from an arbitrary culture-based association between a posture and a word. Through behavioral and neuromodulation studies, investigating how the interaction between the language and motor system could change according to the manual and verbal signals’ conventionalization could help better understand language development. Moreover, longitudinal studies comparing children with developmental language disorder (DLD) or typical development (TD) could help to determine the relation between gesture production and lexicon acquisition (see also [91]).

Lastly, potential application in clinical populations affected by acquired language disorders (aphasia) should be considered, exploiting symbolic gestures to extend the impact of naming recovery based on observation of motor stimuli beyond action verbs [92,93].

## Figures and Tables

**Table 1:** Gestures and words (translated into English) presented in the experiment. The words in Italian are presented in brackets.

Gesture Stimuli	Verbal Stimuli			
Gesture	Congruent Words	Incongruent Words	Pseudowords	
Thumb up	Alright (BENE)	Air (ARIA)	NEBA	RITIA
Thumb Down	Badly (MALE)	Faith (FEDE)	LEMA	DIFE
Stop	Standstill (FERMO)	Idea (IDEA)	MERFO	DEIA

**Table 2:** A summary of the results of post-hoc analysis carried out after the three factor interaction ANOVA (TRAINING × SEMANTICS × STIMULATION DELAY) on normalized FDI MEPs amplitudes. PRE and POST labels indicate pre and post-training phases. The upper side of the table shows the significant comparisons that emerged considering the differences between conditions at each stimulation delay. The lower side shows the significant comparisons that emerged considering the differences between the same stimuli at different stimulation delays. CW: Congruent Words; IW: Incongruent words; CPW: Congruent Pseudowords; IPW: Incongruent Pseudowords.

Significant Comparisons	T1: 100 ms	T2: 250 ms		T3: 500 ms
Within stimulation time (*p* < 0.05)	PRE CW vs.POST IPW PRE CW vs. POST CW PRE IW vs. POST IPW PRE IW vs. POST CW PRE CPW vs. POST IW	PRE CW vs. POST CW PRE IW vs. POST CW		PRE CW vs. POST CW PRE IW vs. POST CW
Between stimulation time (*p* < 0.05)	Congruent Words		Incongruent Words	
	PRE CW 100 vs. PRE CW 500 PRE CW 250 vs. PRE CW 500		PRE IW 100 vs. PRE CW 500 PRE IW 250 vs. PRE CW 500 POST IW 250 vs. POST IW 500	

## Data Availability

The data supporting this study’s findings are available from the corresponding author D.D.M. upon reasonable request.

## References

[1] Paivio A. (1990). Mental Representations: A Dual Coding Approach.

[2] Pecher D., Boot I., Van Dantzig S., Ross B.H. (2011). Chapter Seven—Abstract Concepts: Sensory-Motor Grounding, Metaphors, and Beyond. Psychology of Learning and Motivation.

[3] Shallice T., Cooper R.P. (2013). Is There a Semantic System for Abstract Words?. Front. Hum. Neurosci..

[4] Dove G. (2016). Three Symbol Ungrounding Problems: Abstract Concepts and the Future of Embodied Cognition. Psychon. Bull. Rev..

[5] Borghi A.M., Barca L., Binkofski F., Tummolini L. (2018). Varieties of Abstract Concepts: Development, Use and Representation in the Brain. Philos. Trans. R. Soc. B Biol. Sci..

[6] Buccino G., Colagè I., Silipo F., D’Ambrosio P. (2019). The Concreteness of Abstract Language: An Ancient Issue and a New Perspective. Brain Struct. Funct..

[7] Mkrtychian N., Blagovechtchenski E., Kurmakaeva D., Gnedykh D., Kostromina S., Shtyrov Y. (2019). Concrete vs. Abstract Semantics: From Mental Representations to Functional Brain Mapping. Front. Hum. Neurosci..

[8] Barsalou L.W. (1999). Perceptual Symbol Systems. Behav. Brain Sci..

[9] Barsalou L.W. (2008). Grounded Cognition. Annu. Rev. Psychol..

[10] Zwaan R.A. (2004). The Immersed Experiencer: Toward an Embodied Theory of Language Comprehension. The Psychology of Learning and Motivation: Advances in Research and Theory, Vol. 44.

[11] Gallese V., Lakoff G. (2005). The Brain’s Concepts: The Role of the Sensory-Motor System in Conceptual Knowledge. Cogn. Neuropsychol..

[12] Mahon B.Z., Caramazza A. (2008). A Critical Look at the Embodied Cognition Hypothesis and a New Proposal for Grounding Conceptual Content. J. Physiol.-Paris.

[13] Meteyard L., Cuadrado S.R., Bahrami B., Vigliocco G. (2012). Coming of Age: A Review of Embodiment and the Neuroscience of Semantics. Cortex.

[14] Pulvermüller F. (2013). Semantic Embodiment, Disembodiment or Misembodiment? In Search of Meaning in Modules and Neuron Circuits. Brain Lang..

[15] Fernandino L., Humphries C.J., Conant L.L., Seidenberg M.S., Binder J.R. (2016). Heteromodal Cortical Areas Encode Sensory-Motor Features of Word Meaning. J. Neurosci..

[16] Harpaintner M., Trumpp N.M., Kiefer M. (2020). Time Course of Brain Activity during the Processing of Motor- and Vision-Related Abstract Concepts: Flexibility and Task Dependency. Psychol. Res..

[17] Buccino G., Riggio L., Melli G., Binkofski F., Gallese V., Rizzolatti G. (2005). Listening to Action-Related Sentences Modulates the Activity of the Motor System: A Combined TMS and Behavioral Study. Cogn. Brain Res..

[18] Pulvermüller F., Shtyrov Y., Ilmoniemi R. (2005). Brain Signatures of Meaning Access in Action Word Recognition. J. Cogn. Neurosci..

[19] Dalla Volta R., Fabbri-Destro M., Gentilucci M., Avanzini P. (2014). Spatiotemporal Dynamics during Processing of Abstract and Concrete Verbs: An ERP Study. Neuropsychologia.

[20] Dalla Volta R., Avanzini P., De Marco D., Gentilucci M., Fabbri-Destro M. (2018). From Meaning to Categorization: The Hierarchical Recruitment of Brain Circuits Selective for Action Verbs. Cortex.

[21] Innocenti A., Stefani E.D., Sestito M., Gentilucci M. (2014). Understanding of Action-Related and Abstract Verbs in Comparison: A Behavioral and TMS Study. Cogn Process.

[22] Glenberg A.M., Sato M., Cattaneo L., Riggio L., Palumbo D., Buccino G. (2008). Processing Abstract Language Modulates Motor System Activity. Q. J. Exp. Psychol..

[23] Scorolli C., Binkofski F., Buccino G., Nicoletti R., Riggio L., Borghi A.M. (2011). Abstract and Concrete Sentences, Embodiment, and Languages. Front. Psychol..

[24] Schaller F., Weiss S., Müller H.M. (2017). EEG Beta-Power Changes Reflect Motor Involvement in Abstract Action Language Processing. Brain Lang..

[25] Schaller F., Weiss S., Müller H.M. (2017). “Pushing the Button While Pushing the Argument”: Motor Priming of Abstract Action Language. Cogn. Sci..

[26] De Marco D., De Stefani E., Bernini D., Gentilucci M. (2018). The Effect of Motor Context on Semantic Processing: A TMS Study. Neuropsychologia.

[27] Dreyer F.R., Frey D., Arana S., von Saldern S., Picht T., Vajkoczy P., Pulvermüller F. (2015). Is the Motor System Necessary for Processing Action and Abstract Emotion Words? Evidence from Focal Brain Lesions. Front. Psychol..

[28] Dreyer F.R., Pulvermüller F. (2018). Abstract Semantics in the Motor System?—An Event-Related FMRI Study on Passive Reading of Semantic Word Categories Carrying Abstract Emotional and Mental Meaning. Cortex.

[29] Glenberg A.M., Robertson D.A. (1999). Indexical Understanding of Instructions. Discourse Process..

[30] Glenberg A.M., Kaschak M.P. (2002). Grounding Language in Action. Psychon. Bull. Rev..

[31] Borghi A.M., Barca L., Binkofski F., Tummolini L. (2018). Abstract Concepts, Language and Sociality: From Acquisition to Inner Speech. Philos. Trans. R. Soc. B Biol. Sci..

[32] Borghi A.M., Binkofski F., Borghi A.M., Binkofski F. (2014). The WAT Proposal and the Role of Language. Words as Social Tools: An Embodied View on Abstract Concepts.

[33] Granito C., Scorolli C., Borghi A.M. (2015). Naming a Lego World. The Role of Language in the Acquisition of Abstract Concepts. PLoS ONE.

[34] Cangelosi A., Harnad S. The Adaptive Advantage of Symbolic Theft over Sensorimotor Toil: Grounding Language in Perceptual Categories. http://cogprints.org/2036/.

[35] Liberman A.M., Whalen D.H. (2000). On the Relation of Speech to Language. Trends Cogn. Sci..

[36] Gentilucci M., Corballis M.C. (2006). From Manual Gesture to Speech: A Gradual Transition. Neurosci. Biobehav. Rev..

[37] Passingham R.E. (1993). The Frontal Lobes and Voluntary Action.

[38] Rizzolatti G., Arbib M.A. (1998). Language within Our Grasp. Trends Neurosci..

[39] Petrides M., Pandya D.N. (2009). Distinct Parietal and Temporal Pathways to the Homologues of Broca’s Area in the Monkey. PLoS Biol..

[40] Coudé G., Ferrari P.F., Rodà F., Maranesi M., Borelli E., Veroni V., Monti F., Rozzi S., Fogassi L. (2011). Neurons Controlling Voluntary Vocalization in the Macaque Ventral Premotor Cortex. PLoS ONE.

[41] Corballis M.C. (2012). How Language Evolved from Manual Gestures. Gesture.

[42] Andric M., Solodkin A., Buccino G., Goldin-Meadow S., Rizzolatti G., Small S.L. (2013). Brain Function Overlaps When People Observe Emblems, Speech, and Grasping. Neuropsychologia.

[43] Gentilucci M., Fogassi L., Luppino G., Matelli M., Camarda R., Rizzolatti G. (1988). Functional Organization of Inferior Area 6 in the Macaque Monkey. Exp. Brain Res..

[44] Gentilucci M., Campione G.C. (2011). Do Postures of Distal Effectors Affect the Control of Actions of Other Distal Effectors? Evidence for a System of Interactions between Hand and Mouth. PLoS ONE.

[45] Goldin-Meadow S., Alibali M.W. (2013). Gesture’s Role in Speaking, Learning, and Creating Language. Annu. Rev. Psychol..

[46] Özçalışkan Ş., Goldin-Meadow S. (2005). Gesture Is at the Cutting Edge of Early Language Development. Cognition.

[47] Colonnesi C., Stams G.J.J.M., Koster I., Noom M.J. (2010). The Relation between Pointing and Language Development: A Meta-Analysis. Dev. Rev..

[48] Kuhn L.J., Willoughby M.T., Wilbourn M.P., Vernon-Feagans L., Blair C.B. (2014). Early Communicative Gestures Prospectively Predict Language Development and Executive Function in Early Childhood. Child Dev..

[49] Barsalou L.W. (2010). Grounded Cognition: Past, Present, and Future. Top. Cogn. Sci..

[50] Bernardis P., Gentilucci M. (2006). Speech and Gesture Share the Same Communication System. Neuropsychologia.

[51] Gentilucci M., Bernardis P., Crisi G., Volta R.D. (2006). Repetitive Transcranial Magnetic Stimulation of Broca’s Area Affects Verbal Responses to Gesture Observation. J. Cogn. Neurosci..

[52] De Marco D., De Stefani E., Gentilucci M. (2015). Gesture and Word Analysis: The Same or Different Processes?. NeuroImage.

[53] De Stefani E., De Marco D. (2019). Language, Gesture, and Emotional Communication: An Embodied View of Social Interaction. Front. Psychol..

[54] Capirci O., Contaldo A., Caselli M.C., Volterra V. (2005). From Action to Language through Gesture: A Longitudinal Perspective. Gesture.

[55] Volterra V., Capirci O., Rinaldi P., Sparaci L. (2018). From Action to Spoken and Signed Language through Gesture: Some Basic Developmental Issues for a Discussion on the Evolution of the Human Language-Ready Brain. Interact. Stud..

[56] Kendon A. (1988). How Gestures Can Become like Words. Cross-Cultural Perspectives in Nonverbal Communication.

[57] Shtyrov Y., Butorina A., Nikolaeva A., Stroganova T. (2014). Automatic Ultrarapid Activation and Inhibition of Cortical Motor Systems in Spoken Word Comprehension. Proc. Natl. Acad. Sci. USA.

[58] Barbieri F., Buonocore A., Volta R.D., Gentilucci M. (2009). How Symbolic Gestures and Words Interact with Each Other. Brain Lang..

[59] Mayer K.M., Yildiz I.B., Macedonia M., von Kriegstein K. (2015). Visual and Motor Cortices Differentially Support the Translation of Foreign Language Words. Curr. Biol..

[60] Macedonia M., Mueller K. (2016). Exploring the Neural Representation of Novel Words Learned through Enactment in a Word Recognition Task. Front. Psychol..

[61] Repetto C., Pedroli E., Macedonia M. (2017). Enrichment Effects of Gestures and Pictures on Abstract Words in a Second Language. Front. Psychol..

[62] Mathias B., Sureth L., Hartwigsen G., Macedonia M., Mayer K.M., von Kriegstein K. (2021). Visual Sensory Cortices Causally Contribute to Auditory Word Recognition Following Sensorimotor-Enriched Vocabulary Training. Cereb. Cortex.

[63] Alibali M.W., DiRusso A.A. (1999). The Function of Gesture in Learning to Count: More than Keeping Track. Cogn. Dev..

[64] Ruciński M., Cangelosi A., Belpaeme T. Robotic Model of the Contribution of Gesture to Learning to Count. Proceedings of the 2012 IEEE International Conference on Development and Learning and Epigenetic Robotics (ICDL).

[65] Cangelosi A., Stramandinoli F. (2018). A Review of Abstract Concept Learning in Embodied Agents and Robots. Philos. Trans. R. Soc. B Biol. Sci..

[66] Oldfield R.C. (1971). The Assessment and Analysis of Handedness: The Edinburgh Inventory. Neuropsychologia.

[67] Wassermann E., Epstein C., Ziemann U., Walsh V. (2008). Oxford Handbook of Transcranial Stimulation.

[68] Rossi S., Hallett M., Rossini P.M., Pascual-Leone A. (2009). Safety, Ethical Considerations, and Application Guidelines for the Use of Transcranial Magnetic Stimulation in Clinical Practice and Research. Clin. Neurophysiol..

[69] Villani C., Lugli L., Liuzza M.T., Nicoletti R., Borghi A.M. (2021). Sensorimotor and Interoceptive Dimensions in Concrete and Abstract Concepts. J. Mem. Lang..

[70] Bertinetto P.M., Burani C., Laudanna A., Marconi L., Ratti D., Rolando C., Thornton A.M. (2005). Colfis (Corpus e Lessico di Frequenza Dell’italiano Scritto). https://www.istc.cnr.it/grouppage/colfis.

[71] Pexman P.M., Heard A., Lloyd E., Yap M.J. (2017). The Calgary Semantic Decision Project: Concrete/Abstract Decision Data for 10,000 English Words. Behav. Res..

[72] Brainard D.H. (1997). The Psychophysics Toolbox. Spat. Vis..

[73] Kleiner M., Brainard D.H., Pelli D.G. (2007). What is new in Psychophysics Toolbox. Perception.

[74] Vainiger D., Labruna L., Ivry R.B., Lavidor M. (2014). Beyond Words: Evidence for Automatic Language–Gesture Integration of Symbolic Gestures but Not Dynamic Landscapes. Psychol. Res..

[75] Rizzolatti G., Cattaneo L., Fabbri-Destro M., Rozzi S. (2014). Cortical Mechanisms Underlying the Organization of Goal-Directed Actions and Mirror Neuron-Based Action Understanding. Physiol. Rev..

[76] Rizzolatti G., Sinigaglia C. (2016). The Mirror Mechanism: A Basic Principle of Brain Function. Nat. Rev. Neurosci..

[77] Pulvermüller F., Härle M., Hummel F. (2001). Walking or Talking?: Behavioral and Neurophysiological Correlates of Action Verb Processing. Brain Lang..

[78] Hauk O., Johnsrude I., Pulvermüller F. (2004). Somatotopic Representation of Action Words in Human Motor and Premotor Cortex. Neuron.

[79] Pulvermüller F., Fadiga L. (2010). Active Perception: Sensorimotor Circuits as a Cortical Basis for Language. Nat. Rev. Neurosci..

[80] Kiefer M., Pulvermüller F. (2012). Conceptual Representations in Mind and Brain: Theoretical Developments, Current Evidence and Future Directions. Cortex.

[81] de Vega M., Moreno V., Castillo D. (2013). The Comprehension of Action-Related Sentences May Cause Interference Rather than Facilitation on Matching Actions. Psychol. Res..

[82] Mollo G., Pulvermüller F., Hauk O. (2016). Movement Priming of EEG/MEG Brain Responses for Action-Words Characterizes the Link between Language and Action. Cortex.

[83] Kousta S.-T., Vigliocco G., Vinson D.P., Andrews M., Del Campo E. (2011). The Representation of Abstract Words: Why Emotion Matters. J. Exp. Psychol. Gen..

[84] Vigliocco G., Kousta S.-T., Della Rosa P.A., Vinson D.P., Tettamanti M., Devlin J.T., Cappa S.F. (2014). The Neural Representation of Abstract Words: The Role of Emotion. Cereb. Cortex.

[85] Moseley R., Carota F., Hauk O., Mohr B., Pulvermüller F. (2012). A Role for the Motor System in Binding Abstract Emotional Meaning. Cereb. Cortex.

[86] Fischer M.H., Shaki S. (2018). Number Concepts: Abstract and Embodied. Philos. Trans. R. Soc. B Biol. Sci..

[87] Kiefer M., Barsalou L.W., Prinz W., Beisert M., Herwig A. (2013). Grounding the Human Conceptual System in Perception, Action, and Internal States. Action Science: Foundations of an Emerging Discipline.

[88] Fingerhut J., Prinz J.J. (2018). Grounding Evaluative Concepts. Philos. Trans. R. Soc. B Biol. Sci..

[89] Vukovic N., Shtyrov Y. (2019). Learning with the Wave of the Hand: Kinematic and TMS Evidence of Primary Motor Cortex Role in Category-Specific Encoding of Word Meaning. NeuroImage.

[90] Van Dam W.O., van Dijk M., Bekkering H., Rueschemeyer S.-A. (2012). Flexibility in Embodied Lexical-Semantic Representations. Hum. Brain Mapp..

[91] Lüke C., Ritterfeld U., Grimminger A., Rohlfing K.J., Liszkowski U. (2020). Integrated Communication System: Gesture and Language Acquisition in Typically Developing Children and Children with LD and DLD. Front. Psychol..

[92] Bonifazi S., Tomaiuolo F., Altoè G., Ceravolo M.G., Provinciali L., Marangolo P. (2013). Action Observation as a Useful Approach for Enhancing Recovery of Verb Production: New Evidence from Aphasia. Eur. J. Phys. Rehabil. Med..

[93] Murteira A., Nickels L. (2020). Can Gesture Observation Help People with Aphasia Name Actions?. Cortex.

